# Effect of supplementing sheep diets with macroalgae species on *in vivo* nutrient digestibility, rumen fermentation and blood amino acid profile

**DOI:** 10.1017/S1751731119001502

**Published:** 2019-07-11

**Authors:** Ş. Özkan Gülzari, V. Lind, I. M. Aasen, H. Steinshamn

**Affiliations:** 1 Division of Food Production and Society, Department of Grassland and Livestock, Norwegian Institute of Bioeconomy Research, PO Box 115, 1431 Ås, Norway; 2 SINTEF Industry, 7465 Trondheim, Norway

**Keywords:** *Saccharina latissima*, *Porphyra spp*, digestibility, *in vivo*, ruminant

## Abstract

In this study, a brown macroalgae species, *Saccharina latissima*, processed to increase its protein concentration, and a red macroalgae species, *Porphyra spp*., were used to evaluate their *in vivo* digestibility, rumen fermentation and blood amino acid concentrations. Four castrated rams were used, whose diets were supplemented with a protein-rich fraction of *S. latissima*, a commercial *Porphyra spp*. and soybean meal (SBM). Our results show that the protein digestibility of a diet with *S. latissima* extract was lower (0.55) than those with *Porphyra spp*. (0.64) and SBM (0.66). In spite of the higher nitrogen (**N**) intake of diets containing *Porphyra spp*. and SBM (20.9 and 19.8 g N/day, respectively) than that with *S. latissima* (18.6 g N/day), the ratio of N excreted in faeces to total N intake was significantly higher in the diet with *S. latissima* than those with *Porphyra spp*. and SBM. This reflects that the utilization of protein in *S. latissima* was impaired, possibly due to reduced microbial activity. The latter statement is corroborated by lower volatile fatty acid composition (25.6, 54.8 and 100 mmol/l for *S. latissima, Porphyra spp*. and SBM, respectively) and a non-significant tendency for lower ammonia concentration observed in diets with *S. latissima* and *Porphyra spp*. compared to SBM. It is important to note that the *S. latissima* used in this trial was rinsed during processing to remove salt. This process potentially also removes other water-soluble compounds, such as free amino acids, and may have increased the relative fraction of protein resistant to rumen degradation and intestinal absorption. Furthermore, the phlorotannins present in macroalgae may have formed complexes with protein and fibre, further limiting their degradability in rumen and absorption in small intestines. We recommend that further studies explore the extent to which processing of macroalgae affects its nutritive properties and rumen degradability, in addition to studies to measure the intestinal absorption of these macroalgae species.

## Implications

The protein concentration of seaweed varies greatly from species to species. Those with low-to-medium protein level require processing to remove the salt and to increase protein concentration. This process applied to *Saccharina latissima* species appeared to have reduced the nutrient availability, reflecting lower protein digestibility than that of *Porphyra spp*. and soybean meal. Both rumen fermentation and intestinal absorption were impaired in animals fed *S. latissima*. If the limitations in processing can be fathomed, *S. latissima* may present a potential biomass source that is abundant in nature; however, the challenges in its palatability and chemical composition should be addressed.

## Introduction

Future projections estimate an increase in the import of soybean in Norway by 35% in 2050 compared to 1961 to 1990 levels, due to increasing demand for food and protein feed (Özkan Gülzari *et al*., 2017). This threatens the competitiveness of ruminant production systems and urges the need for alternative protein sources with local origin. For this purpose, macroalgae (also known as seaweed) present a significant and yet potential biomass source. Currently, the brown macroalgae *Saccharina latissima* constitutes the major cultivated species in Europe. Despite its relatively low protein concentration (50–150 g/kg DM), large-scale cultivation may provide significant amounts of protein for use in animal feed. Red macroalgae contain a higher protein concentration than brown macroalgae, but cultivation technology does not yet exist for native, Northern European species. However, red macroalgae (e.g. *Porphyra spp*.) are the largest source of food among macroalgae, for example, nori in Japanese sushi (Makkar *et al*., 2016), and contain up to 347 g/kg DM protein (Tayyab *et al*., 2016).

A number of studies have investigated the digestibility of brown and red macroalgae. An *in vitro* trial measuring the organic matter (**OM**) digestibility of *S. latissima* reported digestibility as high as 0.97 in sheep (Makkar *et al*., 2016). A previous study by Ramin *et al*. (2017) found that increasing the proportion of a protein-enriched fraction of *S. latissima* in an *in vitro* trial increased both the OM digestibility and utilizable protein concentration. *Porphyra spp*. were tested by Tayyab *et al*. (2016) *in situ*, and their crude protein concentration was found to be comparable to oil-seed by-products such as sunflower meal and rapeseed meal. However, given that the aforementioned studies are either *in vitro* or *in situ*, there seems to be a lack of studies investigating the *in vivo* digestibility of macroalgae, and the extent to which they may provide comparable nutrient characteristics to soybean meal (**SBM**) for ruminants. To fill this gap, in this study we evaluated the *in vivo* digestibility, rumen fermentation parameters and amino acid composition in plasma of a diet supplemented with *S. latissima* protein extract or *Porphyra spp*., and compared them with a diet containing SBM. Due to its high salt and iodine-concentration, the biomass of *S. latissima* was exposed to a simple processing by which salt concentration was removed and protein concentration was enriched. The *Porphyra spp*, a commercial product produced in large quantities in Asia, were included for comparison; and an extracted SBM was used as reference.

We hypothesise that the protein digestibility and utilization of diets containing extracted *S. latissima* and purchased *Porphyra spp*. are similar to that of SBM, and better than the diet without any additional protein source.

## Materials and methods

### Experimental design and animals

An *in vivo* digestibility trial was run with four wethers of Norwegian White Sheep, 30 months of age and 80 to 88 kg live body weight, using four diets in a 4×4 Latin square design. The four diets were a control, a diet with protein enriched fraction of *S. latissima* (**SW1**), a diet with *Porphyra spp*. (**SW2**) (CoDo International Limited, Qingdao, China) and a diet with extracted SBM (Champion Soyapellets, Felleskjøpet, Lillestrøm, Norway). The trial was run for four periods from October to November 2017. Each period consisted of 8 days adaptation in individual pens followed by 7 days in individual metabolism crates for daily collection of urine, faeces and feed refusal, if any.

### Feeding

Animals were stalled and fed a control diet containing hay, oats and mineral/vitamin pellets in individual pens for 10 days before the experiment started. Body weight was measured every other day to adjust the maintenance requirements for hay. The DM intake was restricted during the trial and corresponded to maintenance requirements of adult rams in energy as calculated according to the body weight at pre-trial period. Diets with protein enrichment (SW1, SW2 and SBM) were planned to be isocaloric and isonitrogenous (Jarrige, 1988). Adjustments were made to the pre-trial rations where oats and mineral/vitamin pellets were removed to balance the diet. In addition, to increase the palatability of those who refused to eat the macroalgae, the protein feed was mixed with a fixed amount of sugar cane molasses (Felleskjøpet Agri, Sandnessjøen, Norway) at the time of feeding. The control group also received the same amount of molasses. The chemical composition of feed ingredients and the formulation and nutritive value of the individual feed ingredients are presented in Tables 1 and 2, respectively (also see Özkan Gülzari *et al*., 2018a). The SW1 included 3.8 g/kg DM phlorotannins as phloroglucinol, which was not analysed for other feed ingredients. Note that even though the diets were designed to be isonitrogenous, chemical analyses showed that some variation in nitrogen (**N**) concentration existed.

**Table 1:** Chemical composition of individual feed ingredients fed to wethers (g/kg DM unless specified otherwise)

	Feed ingredients									
Item	Hay		Molasses		SW1		SW2		SBM	
	Mean	SD	Mean	SD	Mean	SD	Mean	SD	Mean	SD
Ash	43	(0.5)	132	(0.4)	221	(2.7)	87	(3.7)	77	(19.1)
Kjeldahl-Nitrogen	13.3	(0.33)	10.9	(0.09)	35.8	(0.66)	60.9	(0.50)	82.1	(3.18)
Amino acids	57	(6.1)	20	(1.9)	163	(4.0)	282	(2.4)	468	(25.5)
Crude fat	10.5	(0.79)	2.0	(0.34)	18.5	(3.32)	2.5	(1.06)	16.3	(4.3)
aNDFom^1^	615	(8.6)	0	–	408	(25.1)	431	(13.5)	182	(24.0)
ADF^2^	364	(5.0)	0	–	352	(8.5)	66	(7.0)	90	(5.7)
Crude fibre	308	(4.4)	0	–	152	(2.1)	36	(6.1)	64	(3.9)
Gross energy^3^, MJ/kg DM^4^	19.0	(0.08)	15.7	(0.03)	15.7	(0.05)	18.8	(0.07)	19.5	(0.51)
Iodine, mg/kg DM	–		–		1230		1.5		–	

SW1=processed *S. latissima*; SW2=*Porphyra spp*.; SBM=soybean meal.

1 Neutral detergent fibre assayed with a heat stable amylase and expressed exclusive of residual ash.

2 Acid detergent fibre expressed inclusive of residual ash.

3 Gross energy calculated from gross energy determined with bomb calorimetry of diet ingredients. MJ: megajoule.

4 Dry matter.

**Table 2:** Formulation, chemical analysis and nutritive value of experimental diets fed to wethers averaged over four periods

Item	Experimental diets			
	Control	SW1	SW2	SBM
Ingredients (g/kg DM^1^)				
Hay	961	799	854	888
Protein feed^1^	0	162	108	72
Molasses	39	39	38	40
Sum	1000	1000	1000	1000
Chemical analysis (g/kg DM)				
Organic matter	953	925	949	951
Kjeldahl-Nitrogen	13.2	16.8	18.3	18.1
Amino acids	56	73	80	85
aNDFom^2^	591	557	571	559
ADF^3^	349	347	318	330
Crude fibre	296	271	267	278
Gross energy (MJ/kg DM)^4^	18.9	18.3	18.8	18.9
Iodine (mg/kg DM)	0.04	180	0.20	0.04

SW1=diet containing processed *S. latissima*; SW2=diet containing *Porphyra spp*.; SBM=diet containing soybean meal.

1 Dry matter.

2 Neutral detergent fibre assayed with a heat stable amylase and expressed exclusive of residual ash.

3 Acid detergent fibre expressed inclusive of residual ash.

4 Gross energy calculated from gross energy determined with bomb calorimetry of diet ingredients. MJ: megajoule.

Animals were fed their daily allowance in two equal portions at 0800 and 1600 h. Water was freely accessible through individual drinkers in each pen and metabolism crate. Blood and rumen fluid samples were taken on the last day of each collection period. Blood samples were taken before feeding in the morning, and again 3–4 h after morning feeding. Rumen fluid samples were taken 3–4 h after morning feeding. After sampling for blood at noon, animals were handled back to the stable and were offered their adaptation diet of next period in the afternoon feeding. Any feed refusal was collected every morning during the collection period and weighed, and 10% of the total waste was stored at −18°C until further analyses.

### Preparation and chemical analyses of seaweed species

*Porphyra spp.* were purchased in the form of powder (dried and milled) and was used without further processing. The product was stored in a clean and dry place until use. Cultivated *S. latissima* was harvested at the coast of Sør-Trøndelag County, Norway, in May–June 2016. Seawater was drained, and small stones and other impurities were removed manually. The biomass was stored in plastic bags at −20°C until further processing. To produce the protein-enriched product, 2×375 kg biomass wet weight, 750 kg in total, was milled and washed in water (60°C–70°C) to reduce the salt concentration. Alginate lyase was added for a partial degradation of alginate, to facilitate the subsequent separation by centrifugation. The solid phase (‘sludge’) after centrifugation was dried in a pilot-scale Forberg® Dryer (Forberg International AS, Oslo, Norway) at 40°C (product temperature during drying). The salt concentration in the processed biomass (SW1) was reduced from 440 to 180 g/kg DM and the iodine concentration from 5.9 to 1.2 g/kg DM. The protein concentration as total amino acids was increased from 89 to 186 g/kg DM.

### Collection and storage of samples

Feed samples were collected during the adaptation and collection periods for 15 days in each period by taking a handful of each feed ingredient every day and storing them in plastic boxes, except for hay which was stored in a cardboard box to prevent humidification. At the end of each collection period, 2×100 g samples of SW1, SW2, SBM and 2×200 g samples of hay were prepared. Molasses samples were taken as 2×100 g at the end of each collection period. All feed samples were stored at −18°C until further processing.

Faeces and urine were collected and weighed from each animal on a 24-h basis after feeding each morning and stored at 2°C–3°C until the end of the collection period (7th day). Urine was collected in individual buckets. To each collection bucket for urine, 100 ml sulphuric acid was added before collection started, in order to avoid loss of ammonia. A pH indicator strip non-bleeding pH 0–6.0 (Teststrips, pH, MColorpHast™, product number: 1.09531.0001, VWR International, Merck KGaA, Darmstadt, Germany) was used to measure the acidity of urine every day. After 7 days of collection period, pooled samples of urine and faeces were weighed again as control and 10% of the control weight was taken as a subsample and stored at −18°C until further processing. The rest of the urine and faeces samples were discarded.

At the end of each collection period (7th day), blood and rumen fluid samples were taken from each animal. Blood samples were collected from the jugular vein before (at approximately 0800–0830 h) and approximately 4 h after morning feeding (at approximately 1300–1330 h) to Vacuette® EDTA tubes (product number: 454021, Greiner Bio-One, GmbH, Kremsmünster, Austria). Plasma was separated by centrifugation at 2000 rpm for 20 min, after which aliquots of two samples were removed and pooled into a glass vial to give one sample per wether per period. Plasma was stored at −80°C until further chemical analyses.

Rumen fluid samples were taken within 3–4 h after morning feeding at approximately 1200–1300 h via the esophagus, using two flexible polyvinyl chloride tubes, with diameters of around 3 and 1 cm, respectively, of which the latter was connected to a vacuum pump and sucked approximately 50 ml rumen fluid from each animal. Collected samples of rumen fluid were filtered through an absorbent gauze and pH was measured after filtering using a pH indicator strip non-bleeding pH 7.5–14 (Teststrips, pH, MColorpHast™, product number: 1.09532.0001, VWR International, Merck KGaA, Darmstadt, Germany). Three subsamples of each rumen fluid sample were made for analysing ammonia, lactic acid and volatile fatty acids (**VFAs**) by mixing 4 ml rumen fluid with 4 ml hydrochloric acid (**HCl**) 0.5M; 4 ml rumen fluid with 0.5 ml solution A; and 0.8 ml rumen fluid with 0.5 ml solution A, respectively. Solution A included 20 g/l metaphosphoric and 4 g/l crotonic acids in 0.5M HCl for deprotonization of samples whilst HCl was used to acidify the medium. Any leftover rumen fluid was transferred to glass vials and stored as reserve. Both subsamples and the reserve rumen fluid samples were stored at −80°C until further processing.

### Processing and chemical analyses of samples

Feed samples, except for molasses, were ground to pass 1-mm screen using a Tecator Cyclotec Sample Mill® (Foss Analytical Co., Ltd., Suzhou, China), and were analysed for amino acids and iodine (as described below), and feed composition (Norwegian University of Life Sciences: LabTek, **NMBU**, Ås, Norway). Feed refusal was oven dried for 48 h, weighed and reweighed after storage at ambient temperature for 24 h. Frozen faeces samples were course-ground and freeze-dried for 48 h, using a Labconco FreeZone 4.5 Plus® (Kansas City, Missouri, USA) freeze-drier at a temperature and vacuum ranging between −80°C and −86°C, and 0.52 and 0.97 mbar, respectively. The freeze-dried samples were weighed immediately and again after 24 h storage in ambient temperature. The samples were then ground to pass 1-mm screen using a Tecator Cyclotec Sample Mill® (Foss Analytical Co., Ltd., Suzhou, China). The final weight of the samples was measured, and subsamples were taken and stored in plastic zipped bags at −18°C until further chemical analyses at LabTek, NMBU, for composition.

Feed, feed refusal and faeces samples were analysed for DM (103°C for at least 4 h to constant weight), ash (550°C for at least 4 h), Kjeldahl-N (KjeltecTM 8400; Foss Electric, Hillerød, Denmark), crude fat (accelerated solvent extraction, ASE^TM^ 350 Accelerated Solvent Extractor, Dionex, USA) and gross energy (PARR 1281 Bomb Calorimeter, Moline, Illinois, USA). Neutral detergent fibre was determined with an ANKOM200 fibre analyser (ANKOM Technology, Fairport, New York, USA) according to Mertens (2002) using sodium sulphite, alpha amylase and ash correction (**aNDFom**), and acid detergent fibre (**ADF**) was determined according to Method 973.18 (AOAC, 2000) with the modification that the samples were not washed with acetone and were corrected for ash. Iodine in macroalgae was determined according to Roleda *et al.* (2018) who used the HPLC method to extract iodine by dry alkaline incineration (Nitschke and Stengel, 2015). The polyphenolic concentration in the *S. latissima* extract was determined according to Roleda *et al.* (2019) using phloroglucinol as standard reference.

Amino acid concentration in feed ingredients was analysed by an HPLC system (Agilent Infinity 1260, Agilent Technologies) coupled to an online post-column derivatization module (Pinnacle PCX, Pickering laboratories, Mountain View, California, USA), using ninhydrin (Trione) as a derivatizing reagent and Na+-ion exchange column (4.6×110 mm, 5 µm). Eighteen standard amino acids, ammonia and taurin were quantified from standard curves measured with amino acid standards (Pickering Laboratories, Mountain View, California, USA) (Table 3).

**Table 3:** Amino acid concentration in experimental rations fed to wethers

Item	Experimental diets			
	Control	SW1	SW2	SBM
Essential AA^1^ (g/kg DM^2^)				
Histidine	1.1	1.5	1.6	2.0
Isoleucine	2.8	3.6	3.7	4.3
Leucine	5.0	6.5	6.9	7.3
Lysine	3.3	4.3	4.8	5.4
Methionine	0.6	1.0	0.8	0.8
Phenylalanine	3.3	4.4	4.5	5.1
Threonine	2.2	2.9	3.2	2.7
Tryptophan	0.6	0.5	0.8	0.5
Valine	3.9	5.1	5.9	5.3
Non-essential AA (g/kg DM)				
Alanine	4.5	6.1	7.9	5.8
Arginine	2.8	3.9	4.6	4.9
Aspartic acid	5.4	6.6	6.9	8.7
Cystine	0.6	1.2	2.0	0.8
Glutamic acid	6.7	8.5	8.7	12.3
Glycine	3.3	4.6	4.5	4.4
Proline	0.6	1.2	2.0	0.8
Serine	3.9	5.1	5.6	6.0
Tyrosine	1.7	2.2	2.4	2.6

SW1=diet containing processed *S. latissima*; SW2=diet containing *Porphyra spp*.; SBM=diet containing soybean meal.

1 Amino acid(s).

2 Dry matter.

The frozen urine samples were thawed and subsamples were stored at 4°C until further chemical analyses at Vitas AS (Oslo, Norway) for iodine, and at LabTek, NMBU, for Kjeldahl N (Kjeltec 2460; Foss Electric, Hillerød, Denmark). Urine was diluted and homogenized before the samples were analysed for their iodine concentration by inductively coupled plasma mass spectrometry (Agilent 7900 ICP-MS, Japan). Unknowns were calibrated against known standards from Sigma-Aldrich.

Plasma samples were sent without further processing to University of Helsinki for amino acid analysis. Plasma amino acid concentrations were determined as described by Puhakka *et al*. (2016). Briefly, plasma samples were precipitated using 10% sulphosalisylic acid and analysed by ultra-performance liquid chromatography, equipped with an Ethylene Bridged Hybrid C_18_ column, and a photodiode array detector to detect amino acids.

The three subsamples of rumen fluid per animal and period were sent without further processing to University of Helsinki for ammonia and VFA analyses. Ammonia and VFA from rumen fluid samples were analysed according to McCullough (1967) and Lamminen *et al.* (2017), respectively.

### Statistical analyses

Data were analysed using Proc MIXED in SAS (Statistical Analysis System Institute Inc., 2011) for a 4×4 Latin square design according to the following model:

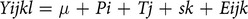

where *Yijkl* is the dependent variable, *µ* the overall mean, *Pi* the effect of period *i*, *Tj* the effect of diet *j*, *sk* the effect of sheep *k* and *Eijk* the residual error. Period and diet were considered fixed effects, using the ‘Repeated’ statement to account for within-animal time-dependent correlations. The optimal covariance structure was assessed for each dependent variable with attention to the corrected Akaike’s information criterion. Degrees of freedom were estimated by using the formula of Satterthwaite. In two out of four periods, the animals refused to eat some of the SW1 supplement. The data from these sheep were excluded from the data analysis (*n* = 14). Differences between least squares means of response variables were estimated with Tukey’s test. Significance was declared at *P* ≤ 0.05 and trends at 0.05<*P* ≤ 0.10. All reported values are least squares means.

## Results

### Total track digestibility

The digestibility of DM, OM, aNDFom, ADF, crude fibre (**CF**) and energy did not differ among the diets (Table 4). Nitrogen digestibility of diets containing SW2 and SBM was similar, which was higher than those of Control and SW1 (*P*=0.002). Nitrogen digestibility of the protein feeds, as calculated by difference, were 0.74, 0.89 and 0.97 for SW1 (SEM: 0.024), SW2 and SBM (SEM: 0.023), respectively. See also Özkan Gülzari *et al*. (2018a and 2018b) for preliminary results presented in conferences.

**Table 4:** Effect of diet on total digestibility in wethers (least square means)

	Experimental diets					
	Control	SW1	SW2	SBM	SEM^5^	*P* value^6^
n	4	2	4	4		
Intake (g DM^1^/day)	1104	1106	1139	1094	0.36/0.55	
Faecal excretion (g DM/day)	352	360	354	337	10.6/16.2	
Digestibility (coefficient)						
Dry matter	0.68	0.67	0.69	0.69	0.0095/0.0146	
Organic matter	0.70	0.69	0.71	0.71	0.0096/0.0146	
Nitrogen	0.55^b^	0.55^b^	0.64^a^	0.66^a^	0.0087/0.0134	**
aNDFom^2^	0.69	0.70	0.70	0.69	0.0122/0.0186	
ADF^3^	0.64	0.62	0.61	0.62	0.0201/0.0306	
Crude fibre	0.68	0.66	0.66	0.68	0.0154/0.0236	
DE/GE^4^	0.66	0.65	0.67	0.67	0.0103/0.0157	

SW1=diet containing processed *S. latissima*; SW2=diet containing *Porphyra spp*.; SBM=diet containing soybean meal.

1 Dry matter.

2 Neutral detergent fibre assayed with a heat stable amylase and expressed exclusive of residual ash.

3 Acid detergent fibre expressed inclusive of residual ash.

4 Ratio of digestible energy/gross energy, where digestible energy was calculated as the difference between gross energy and energy in excreted faeces.

5 Standard error of the mean for Control, SW2 and SBM, and SW1, respectively.

6 **P<0.01.

^a,b^ Values within a row with different superscripts differ significantly at *P*<0.05.

### Nitrogen excretion

Total N intake (g/day) was similar in diets containing SW2 and SBM (20.9 and 19.8, respectively), which was higher than that of SW1. The latter was also found to be higher than that of Control (Table 5). Results show that relatively more N was excreted in faeces than in urine in SW1 than in SW2, SBM and Control diets (*P* = 0.014). Despite the significant difference in urine N excretion, the proportion of urine N excreted of the total N intake did not differ between SW1 and SW2.

**Table 5:** Intake and excretion of nitrogen in urine and faeces of wethers

	Experimental diets					
	Control	SW1	SW2	SBM	SEM^2^	*P* value^3^
n	4	2	4	4		
N^1^ intake (g/day)	14.5	18.6	20.9	19.8		
Faecal N (g/day)	6.6^b^	8.3^a^	7.5^ab^	6.8^b^	0.17/0.26	*
Urine N (g/day)	5.8^c^	8.2^b^	10.6^a^	10.9^a^	0.26/0.39	***
Urine N excreted/total N intake (g/g)	0.40^b^	0.44^ab^	0.51^a^	0.55^a^	0.017/0.026	*
Urine N excreted/digested N (g/g)	0.74	0.79	0.79	0.84	0.033/0.051	
Faecal N excreted/total N intake (g/g)	0.46^a^	0.45^a^	0.36^b^	0.34^b^	0.009/0.013	**
Total N Excreted/total N intake (g/g)	0.85	0.89	0.87	0.90	0.019/0.029	

SW1=diet containing processed *S. latissima*; SW2=diet containing *Porphyra spp*.; SBM=diet containing soybean meal.

1 Nitrogen.

2 Standard error of the mean for Control, SW2 and SBM, and SW1, respectively.

3 * *P*<0.05, ** *P*<0.01, *** *P*<0.001.

^a,b,c^ Values within a row with different superscripts differ significantly at P<0.05.

### Ammonia and volatile fatty acid composition in rumen fluid

The ammonia concentration in the rumen fluid tended (*P* = 0.05) to be highest in the diet containing SBM (7.36 mmol/l) and lowest in the diet containing SW1 (2.29 mmol/l). Urea concentration was similar in diets containing SW1, SW2 and SMB, and significantly higher in SW2 and SBM than in Control. There was a strong effect of diet on rumen fermentation. The SW1 reduced the rumen fermentation greatly compared to SBM, where VFA profiles were 25.6 and 100 mmol/l, respectively. Nevertheless, the fermentation pattern was not affected, as the molar proportions of acetic acid, propionic acid and butyric acid compositions (mmol/mol) did not differ among diets (Table 6).

**Table 6:** Rumen fermentation products of wethers fed on four different diets

	Experimental diets					
	Control	SW1	SW2	SBM	SEM^2^	*P* value^3^
n	4	2	4	4		
Ammonia, mmol/l	3.44	2.29	3.47	7.36	0.80/1.22	(*)
VFA^1^ total, mmol/l	70.0^ab^	25.6^b^	54.8^b^	100^a^	15.1/20.0	*
VFA proportion, mmol/mol						
Acetic acid	701	707	707	705	6.8/10.3	
Propioic acid	173	151	151	158	7.7/11.3	
Butyric acid	85	93	96	93	6.1/8.6	
Isobutyric acid	3.0	4.4	3.9	5.1	0.76/1.17	
Isovaleric acid	2.9	4.0	3.6	4.8	0.74/1.14	
Valeric acid	5.6	5.6	5.9	6.3	0.56/0.85	
Caproic acid	29	33	32	27	3.4/5.1	
Acetic : Propinoic (ratio)	4.1	4.7	4.7	4.5	0.19/0.28	
pH	8.0	8.3	8.6	7.8	0.24/0.36	

SW1=diet containing processed *S. latissima*; SW2=diet containing *Porphyra spp*.; SBM=diet containing soybean meal.

1 Volatile fatty acids.

2 Standard error of the mean for Control, SW2 and SBM, and SW1, respectively.

3 (*) P<0.10 and * P<0.05.

^a,b^ Values within a row with different superscripts differ significantly at *P*<0.05.

### Amino acid composition in blood plasma

The diet composition had only negligible effect on plasma amino acid levels (Table 7). The plasma concentration of 1-Methyl-Histidine (**1MH**) was higher with the control diets than with the diets with extra protein. The concentration of methionine, glutamate and α-aminobutyric acid tended (*P* < 0.10) to be higher in SW1 than in SW2 and SBM diets.

**Table 7:** Plasma amino acid composition of wethers fed on four different diets

	Experimental diets					
	Control	SW1	SW2	SBM	SEM^2^	*P* value^3^
n	4	2	4	4		
Essential AA^1^ (µmol/l)						
Histidine	54	52	46	52	3.6/5.5	
Isoleucine	88	80	76	82	7.2/11.1	
Leucine	100	104	92	89	7.5/9.6	
Lysine	126	114	106	107	16.8/23.6	
Methionine	19	19	16	17	1.4/1.6	(*)
Phenylalanine	40	42	44	38	2.1/2.8	
Threonine	117	152	93	111	13.1/20.0	
Tryptophan	31	34	32	31	1.5/2.3	
Valine	199	187	181	186	15.4/23.5	
Non-essential AA (µmol/l)						
Arginine	139	140	145	150	23.4/31.7	
Alanine	264	271	194	210	13.3/33.6	
Aspartic acid	7.5	9.1	6.4	7.6	0.96/1.15	
Glutamic acid	78	89	61	67	5.3/9.9	(*)
Glutamine	335	353	288	346	31.6/48.3	
Glycine	438	347	310	407	40.4/61.7	
Proline	81	82	65	73	7.6/10.8	
Serine	79	72	56	74	7.0/10.7	
Citrulline	129	118	116	128	18.1/24.8	
Ornithine	73	80	62	69	8.5/11.9	
Taurine	53	56	61	43	3.2/4.8	(*)
Tyrosine	56	58	48	53	4.3/6.6	
Hydroxyproline	20	20	17	19	2.1/3.0	
3-Methylhistidine	6.8	7.8	6.3	6.5	0.51/0.60	
1-Methylhistidine	78^a^	64^b^	65^b^	65^b^	21.3/21.5	*
Aspartic acid	7.5	9.1	6.4	7.6	0.96/1.15	
Sarcosine	7.0	7.4	6.3	6.5	0.56/0.78	
β-Alanine	2.8	2.8	2.6	2.6	0.11/0.16	
α-aminoadipic acid	5.2	3.9	4.0	4.1	0.66/0.75	(*)
α-amino-N-butric acid	12.2	15.0	9	11	1.4/1.9	(*)
Cystathionine	3.9	5.4	4.3	4.1	0.67/0.89	
Cystine	8.0	10.0	8.5	8.0	1.3/1.9	
Essential amino acids	894	887	815	848	79.2/118.2	
Essential amino acids/total amino acids (ratio)	0.332	0.341	0.362	0.338	0.0061/0.0092	(*)

SW1=diet containing processed *S. latissima*; SW2=diet containing *Porphyra spp*.; SBM=diet containing soybean meal.

1 Amino acid(s).

2 Standard error of the mean for Control, SW2 and SBM, and SW1, respectively.

3 (*) *P*<0.10 and *P<0.05.

^a,b^ Values within a row with different superscripts differ significantly at *P*<0.05.

## Discussion

### Total tract digestibility and N excretion

The low N digestibility in SW1 compared to SW2 and SBM can be explained by both the impaired rumen fermentation and the limited absorption of rumen-undegradable-containing compounds in the small intestines.

Macroalgae contain phlorotannins, phenolic compounds forming insoluble complexes with protein, whereby preventing protein degradation (Burtin, 2003). Phlorotannins are known to reduce digestion (Arnold and Targett, 1998). Wang *et al*. (2009) studied the effects of phlorotannins extracted from brown macroalgae on the rumen bacterial population and fermentation, and found that phlorotannins inhibited the growth of *Fibrobacter succinogenes* and increased the non-cellulolytic bacteria *Selenomonas ruminantium*, *Ruminobacter amylophilus* and *Prevotella bryantii*. A study by Gaillard *et al*. (2018) reported that low ruminal degradability of amino acids of *Laminaria*, a brown seaweed genus related to *Saccharina*, was probably associated with phlorotannins. We did not analyse the phlorotannins in *Porphyra spp*., but the 3.8 g/kg DM phlorotannins in SW1 may have bound and limited the digestibility of protein (Clark *et al*., 1987). By binding the proteins and carbohydrates, tannins may leave less of the nutrients available for rumen microbiota, but also inhibit microbial enzyme activity, or directly affect rumen microorganisms, resulting in reduced rumen degradation (Frutos *et al*., 2004).

Poor carbohydrate utilization of bacteria may have further compromised the digestibility of nutrients in animals fed SW1. Given that the soluble fraction is more rapidly degraded in the rumen than the insoluble fraction (Clark *et al*., 1987), it can be interpreted that less of the N in the SW1 was in the soluble fraction than that of SW2 and SBM.

Tannin-protein complexes may have been formed in the rumen pH 3.8–8, but they would have been expected to dissociate in the abomasum where pH levels reduce below 3.5 or in duodenum where pH is above 8 (Frutos *et al*., 2004). If this is the reason for the rumen-undegraded N not to be absorbed in the small intestines in our study, this may be because the tannins interact with the membrane proteins of intestinal mucosa (Frutos *et al*., 2004). This could be investigated by the mobile nylon-bag N disappearance technique (Frydrych, 1992). However, the physical properties of powder form SW1 and SW2 would result in loss of biomass not because they would be ingested but because they would pass through the pores of the nylon bag.

Nitrogen digestibility of SW2 was similar to that of SBM, due possibly to its high rumen degradability and high absorption of rumen-escapable proteins in the small intestine. An earlier study by Tayyab *et al*. (2016) suggests for *Porphyra spp*. that digestibility of escape protein is quite high, and 50% of the protein is degraded in the small intestine. It can be noted that similar amino acid composition in blood plasma for all diets may indicate that the quality of protein was similar among different diets, and that it mainly consisted of microbial protein. The latter statement is based on the fact that the control diet did not include any additional protein source.

Higher ash concentration in SW1 (221 g/kg DM) than in SW2 and SBM may also have affected the digestibility figures. Macroalgae with high concentrations of iodine and ash (e.g. SW1) need to be added to the diet at a low rate, but low inclusion may thwart their positive effects. Arieli *et al*. (1993) reported that supplementing sheep diets with *Ulva* seaweed species gave rise to reduced concentration of digested energy at 9.1 megajoule: **MJ**/kg DM, due mainly to the high ash concentration of seaweed (207 g/kg). It reduces its nutritive value, and therefore its potential as a ruminant protein supplement (Arieli *et al*., 1993). However, the digestible energy concentration of different diets did not differ in this study and was on average 13.8 MJ/kg DM, due possibly to the low inclusion rate in the diet. The ash concentrations of the diets in this study were much lower than the 105.5 g/kg DM of a diet containing *Ulva* species in the study of Arieli *et al*. (1993), which seems to explain the higher digestibility of the diet in our study than in that of Arieli *et al*. (1993). To the best of authors’ knowledge, there are no *in vivo* studies generating data where *S. latissima* was fed to sheep or ruminants, but the high iodine concentration may explain the resistance to consume the SW1 by two of the animals, even though the iodine concentration was significantly reduced (from 6 to 1.2 g/kg DM) by processing.

Reduced bacterial activity was probably the reason for the lower ammonia levels in SW1 than in SW2 and SBM in this study; however, the difference was not significant. Clark *et al*. (1987) stated that feeding cows with alimentary protein sources that were not easily degraded in the rumen may result in low ammonia, amino acids and peptides for ruminal microbiota. Low ammonia concentration as well as a ratio of 0.9 : 1.02 for the excretion of N in urine as a fraction of digested N in sheep fed *Ulva* seaweed species was reported to indicate low rumen degradability of the protein concentration in seaweed (Arieli *et al*., 1993). A lower ratio (0.79) of the N excreted in urine compared to total N intake (g/g) in the current study than that reported by Arieli *et al*. (1993) may be due to the difference in protein level in the diet (low but sufficient protein concentration in our study).

Higher production of VFAs in SBM than in the diets supplemented with seaweed suggests rapid and extensive fermentation in the rumen. Even though the difference was not significant, lower total VFA profile in SW1 than SBM indicated that the predation of rumen protozoa on bacteria was altered, causing more of the bacteria to leave the rumen and therefore reducing microbial N per unit of apparently digested OM (Clark *et al*., 1987).

Clark *et al*. (1987) calculated that 3.7 g of methionine and 18.4 g of lysine from microbial protein escape the rumen for every kg of OM fermented in the rumen. It is important to note that higher concentrations of amino acids present in the diet do not always translate into greater amounts of amino acids passing to the small intestines. Eventually, what determines the amount of amino acids passing to the small intestines is the amino acid concentration of protein that escapes ruminal fermentation (Clark *et al*., 1987). In our study, even though greater amounts of lysine and methionine were detected in plasma for the SW1 than for SW2 and SBM, the difference was not significant. Despite the higher concentrations of lysine in diets containing SW2 and SBM than in SW1, greater lysine concentrations in blood plasma of SW1 indicate that the utilization of lysine in SW1 was hindered. This is further discussed by Gaillard *et al*. (2018) who reported that methionine and lysine in a brown seaweed species *Laminaria* were not degraded in the rumen, although they became available in the small intestines.

### Seaweed harvesting time and processing on digestion parameters

Several factors have an impact on the protein concentration of seaweed. For the brown macroalgae, the protein concentration is highest in winter and early spring, before onset of the accumulation of storage carbohydrates during late spring and summer. Maximum protein coincides with maximum salt (ash) concentration (Schiener *et al*., 2015). The *S. latissima* used in the current work was harvested in May–June with approximately 10% DM and contained almost no laminaran, but ~10% mannitol. In late summer or autumn, the dry weight is ~20%, and the soluble carbohydrates (laminaran and mannitol) constitute more than 30% of DM, with correspondingly lower protein and ash concentrations.

Cultivated seaweeds are harvested in spring, due to fouling and degradation during the summer. Processing to reduce their salt concentration will therefore be required for a high inclusion rate in animal feed. This processing also reduces the concentration of nutrients, such as free amino acids and other water-soluble N-containing compounds.

### Adaptation period as a barrier

The adaptation period before the collection may have been a factor affecting the low digestibility. It is important to note that the carry-over effects between periods were modelled in the statistical analyses. As opposed to the 14-day adaptation period in Carvalho *et al*. (2005) and Milis *et al*. (2005), in this study the rams were adapted to the diet for 8 days. A longer adaptation period may affect the adaptation of rumen microbiota to the new diet, resulting in increased levels of rumen fermentation. However, since the rumen microbiota responds to the diet changes differently, a longer adaptation period is not always justified. An example to this is the study by Fernando *et al*. (2010) who found significantly high number of *Megasphaera elsdenii*, *Streptococcus bovis*, *Selenomonas ruminantium* and *Prevotella bryantii*, but gradually reduced populations of *Butyrivibrio fibrisolvens* and *Fibrobacter succinogenes* when animals were adapted to high-concentrate diet for 7 days. Similarly, in a review on *in vivo* measurement of forage digestibility, Rymer (2000) indicates that animals need 4–12 days to adapt to diets, and that normally 6–8 days would be required (Omed, 1986). Further, Nicholson *et al*. (1956) reported that an adaptation period of 7 days would suffice when a constant hay to concentrate ratio was maintained in spite of a varying protein level. It can be also noted that the ability of protozoon in the rumen to adapt to the seaweed is also associated with the genetics of the animals (Orpin *et al*., 1985). However, given that some sheep, for example, Orkney breed on North Ronaldsay in the United Kingdom, graze principally on seaweed, the ability to degrade seaweed by Orkney sheep renders the adaptation of the microbial population as more of a factor than genetics (Greenwood *et al*., 1983). When decisions are made to increase the length of adaptation, a further consideration should be given to the trade-offs between the expected results and the compromise made for animal welfare, as well as the cost of an additional day in the adaptation.

## Software and data repository resources

None of the data were deposited in an official repository.

## References

[ref32] AOAC 2000 Official methods of analysis. 17th edition. Association of Official Analytical Chemists, Arlington, VA.

[ref1] Arieli A, Sklan D and Kissil G 1993 A note on the nutritive value of *Ulva lactuca* for ruminants. Animal Science 57, 329–331.

[ref2] Arnold TM and Targett NM 1998 Quantifying in situ rates of phlorotannin synthesis and polymerization in marine brown algae. Journal of Chemical Ecology 24, 577–595.

[ref3] Burtin P 2003 Nutritional value of seaweeds. Electronic Journal of Environmental, Agricultural and Food Chemistry 2, 498–503.

[ref4] Carvalho LPF, Melo DSP, Pereira CRM, Rodrigues MAM, Cabrita ARJ and Fonseca AJM 2005 Chemical composition, *in vivo* digestibility, N degradability and enzymatic intestinal digestibility of five protein supplements. Animal Feed Science and Technology 119, 171–178.

[ref5] Clark JH, Murphy MR and Crooker BA 1987 Supplying the protein needs of dairy cattle from by-product feeds. Journal of Dairy Science 70, 1092–1109. 3298341 10.3168/jds.S0022-0302(87)80116-9

[ref6] Fernando SC, Purvis HT, Najar FZ, Sukharnikov LO, Krehbiel CR, Nagaraja TG, Roe BA and DeSilva U 2010 Rumen microbial population dynamics during adaptation to a high-grain diet. Applied and Environmental Microbiology 76, 7482–7490. 20851965 10.1128/AEM.00388-10 PMC2976194

[ref7] Frutos P, Hervás G, Giráldez FJ and Mantecón AR 2004 Tannins and ruminant nutrition. Spanish Journal of Agricultural Research 2, 191–202.

[ref8] Frydrych Z 1992 Intestinal digestibility of rumen undegraded protein of various feeds as estimated by the mobile bag technique. Animal Feed Science and Technology 37, 161–172.

[ref9] Gaillard C, Bhatti HS, Novoa-Garrido M, Lind V, Roleda MY and Weisbjerg MR 2018 Amino acid profiles of nine seaweed species and their *in situ* degradability in dairy cows. Animal Feed Science and Technology 241, 210–222.

[ref10] Greenwood Y, Orpin CG and Paterson IW 1983 Digestibility of seaweeds in Orkney sheep. Journal of Physiology 343, 120.

[ref11] Jarrige R 1988 Alimentation des bovins, ovins & caprins. INRA-Quae, Versailles, France.

[ref12] Lamminen M, Halmemies-Beauchet-Filleau A, Kokkonen T, Simpura I, Jaakkola S and Vanhatalo A 2017 Comparison of microalgae and rapeseed meal as supplementary protein in the grass silage based nutrition of dairy cows. Animal Feed Science and Technology 234, 295–311.

[ref13] Makkar HPS, Tran G, Heuzé V, Giger-Reverdin S, Lessire M, Lebas F and Ankers P 2016 Seaweeds for livestock diets: a review. Animal Feed Science and Technology 212, 1–17.

[ref14] McCullough H 1967 The determination of ammonia in whole blood by a direct colorimetric method. Clinical Chimica Acta; International Journal of Clinical Chemistry and Diagnostic Laboratory Medicine 17, 297–304. 10.1016/0009-8981(67)90133-7 6040460

[ref15] Mertens DR 2002 Gravimetric determination of amylase-treated neutral detergent fiber in feeds with refluxing in beakers or crucibles: collaborative study. Journal of AOAC International (The Association of Analytical Communities) 85, 1217–1240. 12477183

[ref16] Milis C, Liamadis D, Karalazos A and Dotas D 2005 Effects of main protein, non-forage fibre and forage source on digestibility, N balance and energy value of sheep rations. Small Ruminant Research 59, 65–73.

[ref17] Nicholson J, Haynes E, Warner R and Loosli J 1956 Digestibility of various rations by steers as influenced by the length of preliminary feeding period. Journal of Animal Science 15, 1172–1179.

[ref33] Nitschke U and Stengel DB 2015 A new HPLC method for the detection of iodine applied to natural samples of edible seaweeds and commercial seaweed food products. Food Chemistry 172, 326–334. 25442561 10.1016/j.foodchem.2014.09.030

[ref18] Omed HM 1986. Studies of the relationships between pasture type and Quality and the feed intake of grazing sheep. PhD thesis, University College of North Wales, Nagor, UK.

[ref19] Orpin CG, Greenwood Y, Hall FJ and Paterson IW 1985 The rumen microbiology of seaweed digestion in Orkney sheep. Journal of Applied Microbiology 58, 585–596. 10.1111/j.1365-2672.1985.tb01715.x 4030526

[ref20] Özkan Gülzari Ş, Åby BA, Persson T, Höglind M and Mittenzwei K 2017 Combining models to estimate the impacts of future climate scenarios on feed supply, greenhouse gas emissions and economic performance on dairy farms in Norway. Agricultural Systems 157, 157–169.

[ref21] Özkan Gülzari Ş, Lind V, Aasen IM and Steinshamn H 2018a. *In vivo* nutrient digestibility of a protein fraction extracted from macroalgae *Saccharina latissima* in sheep. In Proceedings of the 9th Nordic Feed Science Conference, 12–13 June 2018, Swedish University of Agricultural Sciences, Sweden, pp. 83–86.

[ref22] Özkan Gülzari Ş, Lind V, Aasen IM and Steinshamn H 2018b. Chemical composition and *in vivo* digestibility of seaweed as a protein source for ruminant nutrition. Presented at the 69th Annual Meeting of the European Federation of Animal Science, 27–31 August 2018, Dubrovnik, Croatia, p. 632.

[ref23] Puhakka L, Jaakkola S, Simpura I, Kokkonen T and Vanhatalo A 2016 Effects of replacing rapeseed meal with fava bean at 2 concentrate crude protein levels on feed intake, nutrient digestion, and milk production in cows fed grass silage-based diets. Journal of Dairy Science 99, 7993–8006. 27522411 10.3168/jds.2016-10925

[ref24] Ramin M, de Oliveira Franco M, Roleda MY, Aasen IM, Hetta M and Steinshamn H 2017 Effect of extracted seaweed protein fractions on estimated utilizable crude protein, methane emission and fermentation parameters an *in vitro* evaluation. Proceedings of 8th Nordic Feed Science Conference, 13–14 June 2017, Swedish University of Agricultural Sciences, Sweden, pp. 65–70.

[ref25] Roleda MY, Marfaing H, Desnica N, Jónsdóttir R, Skjermo J, Rebours C and Nitschke U 2019 Variations in polyphenol and heavy metal contents of wild-harvested and cultivated seaweed bulk biomass: health risk assessment and implication for food applications. Food Control 95, 121–134.

[ref26] Roleda MY, Skjermo J, Marfaing H, Jónsdóttir R, Rebours C, Gietl A, Stengel DB and Nitschke U 2018 Iodine content in bulk biomass of wild-harvested and cultivated edible seaweeds: inherent variations determine species-specific daily allowable consumption. Food Chemistry 254, 333–339. 29548461 10.1016/j.foodchem.2018.02.024

[ref27] Rymer C 2000 The measurement of forage digestibility *in vivo* In Forage evaluation in ruminant nutrition (ed. DI Givens, E Owen, RFE Axford, HM Omed), pp. 113–134. CABI Publishing, London, UK.

[ref28] Schiener P, Black KD, Stanley MS and Green DH 2015 The seasonal variation in the chemical composition of the kelp species *Laminaria digitata*, *Laminaria hyperborea*, *Saccharina latissima* and *Alaria esculenta*. Journal of Applied Phycology 27, 363–373.

[ref29] Statistical Analysis System Institute Inc 2011. SAS/STAT® 9.3 user’s guide. Cary, NC, USA.

[ref30] Tayyab U, Novoa-Garrido M, Roleda MY, Lind V and Weisbjerg MR 2016 Ruminal and intestinal protein degradability of various seaweed species measured *in situ* in dairy cows. Animal Feed Science and Technology 213, 44–54.

[ref31] Wang Y, Alexander TW and McAllister TA 2009 *In vitro* effects of phlorotannins from *Ascophyllum nodosum* (brown seaweed) on rumen bacterial populations and fermentation. Journal of the Science of Food and Agriculture 89, 2252–2260.

